# Epidemiology and global spread of emerging tick-borne Alongshan virus

**DOI:** 10.1080/22221751.2024.2404271

**Published:** 2024-09-11

**Authors:** André Gömer, Arthur Lang, Saskia Janshoff, Joerg Steinmann, Eike Steinmann

**Affiliations:** a Department for Molecular und Medical Virology, Ruhr University Bochum, Bochum, Germany; b Institute of Clinical Hygiene, Medical Microbiology and Infectiology, General Hospital Nuremberg, Paracelsus Medical University, Nuremberg, Germany; c Institut für Laboratoriums- und Transfusionsmedizin, Herz- und Diabeteszentrum Nordrhein-Westfalen, Universitätsklinik der Ruhr-Universität Bochum, Bad Oeynhausen, Germany; d Institute of Medical Microbiology, University Hospital of Essen, Essen, Germany

**Keywords:** Alongshan virus, segmented flavi-like viruses, ticks, epidemiology, one health, urbanization, zoonosis, climate change

## Abstract

The emergence and spread of novel viral pathogens is a major threat to human health, particularly in the context of climate and human-induced change in land use. Alongshan virus (ALSV) is a tick-borne virus associated with human disease, which was first identified in northeast China. More recently, several studies reported the emergence of ALSV in mammalian and arthropod hosts in multiple different countries outside of Asia, and the first viral genome sequencing data has become available. ALSV is a member of the Jingmenvirus group closely related to the *Flaviviridae* family. Unusually, the positive-sense, single-stranded RNA genome of ALSV is segmented and consists of four distinct segments, two of which show homology with the NS3 and NS5 protein encoding regions of non-segmented flaviviruses. Transmission of arthropod-borne pathogens will likely increase in the future due to environmental change mediated by a variety of environmental and ecological factors and increasing human encroachment into wild animal habitats. In this review, we present current knowledge of global ALSV distribution and emergence patterns, highlight genetic diversity, evolution and susceptible species. Finally, we discuss the role of this emerging tick-borne virus in the context of urbanization and global health.

## Introduction

According to the World Health Organization, vector-borne diseases make up more than 17% of infectious diseases worldwide and account for more than 700.000 deaths per year (Source: WHO, see https://www.who.int/news-room/fact-sheets/detail/vector-borne-diseases). As these pathogens and their respective vectors can spread dynamically and rapidly, they constitute a considerable burden and threat to global health. In addition, the spread of vector-borne diseases is fuelled by habitat changes caused by climate change or anthropogenic factors and by human encroachment into wildlife habitats. Amongst the pathogens with increased risk for causing human pandemics are Flaviviruses.

*Flaviviridae* are a family of small enveloped viruses with positive-sense RNA genomes of approximately 9.0–13 kb [1]. Known for their high genomic and phenotypic variability, members of the *Flaviviridae* family infect a wide range of mammals, birds, and arthropods and thus have a high socio-economic impact. This family can be divided into four genera with distinct characteristics: Hepacivirus, Pegivirus, Pestivirus and Orthoflavivirus. The majority of members of the genus *Orthoflavivirus* are arthropod-borne and represent important human and veterinary pathogens (*e.g.* yellow fever virus, dengue virus, West Nile virus) [2,3]. Despite the substantial sequence divergence between the genera within the *Flaviviridae*, these viruses exhibit a similar genomic structure characterized by a single open reading frame (ORF) flanked by 5′- and 3′-terminal non-coding regions (NCRs). Structural and nonstructural viral proteins are synthesized as part of a polyprotein that is co- and post-translationally cleaved by viral and cellular proteases. Among the nonstructural protein products, NS3 and NS5 encode the enzymatic domains essential for RNA capping and genome replication, whereas the NS3 and NS2b proteins form a two-component serine protease involved in posttranslational cleavage of the viral polyprotein [4,5]. In 2014, the textbook knowledge of the *Flaviviridae* genomic structure had been revised, when Qin et al. reported the discovery of the first segmented flavi-like virus in ticks, which was subsequently named Jingmen tick virus (JMTV) [6]. JMTV was shown to have a plus-stranded RNA genome composed of four distinct fragments: S1, S2, S3, and S4. Fragments S1 and S3 encode nonstructural proteins (NSP1 and NSP2), which are homologous to NS3 and NS5 of other flaviviruses. The fragments S2 and S4 encode structural proteins (VP1, VP2, and VP3), which present no homology with any known viral sequence outside the Jingmenvirus group to date [6,7]. JMTV has been detected in several tick species as well as mosquitos, rodents, cattle, goats, bats and primates [8]. More recently, JMTV genome sequences were detected in human fatal cases of Crimean-Congo haemorrhagic fevervirus infection in Kosovo [9] and in four individuals from China [10]. These patients presented with an itchy or painful eschar at the site of tick bite including fever, headache, and myalgia, demonstrating that JMTV can infect humans and manifest clinically. As a result of tick-borne disease surveillance in several countries, another unknown virus, termed Alongshan virus (ALSV), has recently been identified in a patient with unexplained febrile illness in Inner Mongolia in 2017 [11]. This virus demonstrated a high sequence similarity to JMTV and has since then been detected in multiple mammals and arthropods and in additional countries across the globe. In this review, we will provide an overview of the current knowledge on ALSV, including its circulation and genetic diversity. Furthermore, we will discuss zoonotic features of ALSV and its impact on global health in the context of urbanization and global warming.

## Alongshan virus circulation

**Figure 1.**
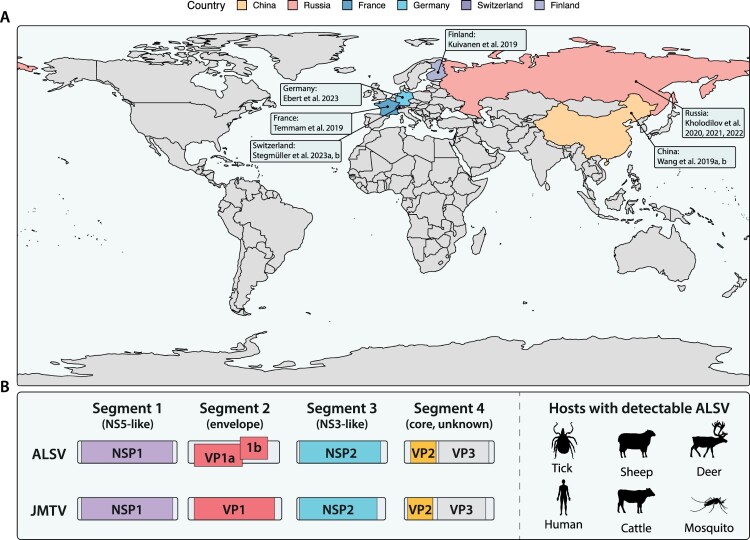
(a) Alongshan virus (ALSV) epidemiology. 272 sequences were downloaded from NCBI, including metadata from sampling locations. In conjunction with reports of ALSV prevalence across Asia and Europe, we summarize in which countries the virus has been detected. (b) Flavivirus particle structure and ALSV and Jingmentick virus genome organization. The genomes are divided into four segments (S1–S4): S1 encodes the NS5-like protein, segment 2 encodes the glycoproteins – monocistronic for JMTV and bicistronic for ALSV, S3 encodes an NS3-like protein and S4 encodes the capsid and the VP3 protein. Icons have been retrieved from phylopic.org.

Since their first description in 2014 [6], segmented flavi-like viruses have been identified worldwide in a variety of hosts [8,12]. Like most Orthoflaviviruses and other members of the Jingmenvirus group, ALSV has been identified in samples obtained from arthropods and various mammals, such as deer, equids and humans across Asia (China and Russia) and Europe (Finland, France, Germany, and Switzerland – Figure 1(a), Table 1). In contrast to JMTV, however, ALSV has not yet been detected in Africa, Oceania, or the Americas – most likely because of lack in surveillance. The first descriptions of ALSV occurred in China – in the Inner Mongolia and Heilongjiang provinces in 2017 [11,13]. Following the index cases, the authors were able to detect ALSV by qPCR in 86 out of 374 patients (∼23%) with an unknown cause of illness and tick-bite history in China. Additionally, these patients did not present indications for other tick-borne infectious diseases including severe fever with thrombocytopenia syndrome virus (SFTSV), Tick-borne Encephalitis Virus (TBEV), Borrelia burgdorferi, Borrelia miyamotoi, anaplasmosis, babesiosis, or rickettsiosis. Using qPCR-based pool testing of ticks and mosquitoes, they were able to show that up to 30 percent of mosquito and 6.5 percent of tick pools tested positive for viral RNA. In a follow-up study, Wang et al. found ALSV in livestock (sheep and cattle) by detecting viral RNA via qPCR and anti-ALSV antibodies using an VP2-based ELISA [13]. Subsequently, ALSV genomic sequences have been detected in Finland [14], Russia [15–17], Serbia [18], Germany [19], Switzerland [20,21], and France [22]. From these studies, however, it remains difficult to conclude how prevalent ALSV is, given the different diagnostic strategies used. Detection of ALSV has been achieved by metagenomic NGS in ticks [20–22]. More frequently, however, ALSV nucleic acid quantification was performed. Here, the rate of RNA-positive animals varied depending on host reservoir, region, and publication: In tick pools from Russia, the prevalence was 0.6–11.1% (calculated as Minimal Infection Rate, MIR) [16]. In questing tick pools from China and Germany, the prevalence ranged between 3.7 and 6.8% [11,19]. In tick pools collected from animals in Germany, the prevalence was 46% [19]. In addition, a cohort of 374 individuals from northern China exhibited up to 23% positivity for ALSV RNA [11]. To date, these patients represent the only confirmed cases of ALSV in humans. Furthermore, ALSV RNA was also detected in different mosquito species (Aedes, Anopheles, Culex) using qPCR pool testing, with up to 30 percent positive pools [11]. Anti-ALSV antibody levels were measured using either an in-house ELISA against the VP2 protein, a Luciferase Immunoprecipitation Assay System (LIPS) against the core protein, or an immunofluorescence assay using Vero E6 cells transfected with VP1a, VP1b and the core protein. These techniques have been employed in multiple studies to detect antibodies associated with ALSV. For instance, Wang et al. (2019) demonstrated that 19 of 86 RNA-positive patients from China exhibited measurable antibodies using an immunofluorescence assay. Furthermore, all 19 serum samples demonstrated neutralizing capacity as determined by a microneutralization assay [11]. In a second study conducted in Finland, 283 patients tested negative for anti-ALSV antibodies [14]. In animals, anti-ALSV antibodies were detected in cattle (∼4.5%) and sheep (∼9%) using VP2 ELISA [13], and in deer (∼3.4%), goats (∼6%), sheep (∼3.6%) and equids (∼14%) by performing an anti-core LIPS [19]. It is important to acknowledge that in the detection of flaviviruses, the presence of cross-reactive antibodies can interfere with accurate diagnosis possibly due to shared sequence and structural homology between these viruses [23–25]. The extent of such interference for members of the Jingmen virus group warrants further investigation. In addition to the antibody quantification methods described above, neutralization assays have been developed that necessitate the use of cell lines exhibiting susceptibility to ALSV infection. The *in vitro* infectivity of ALSV has been demonstrated in numerous studies utilizing a variety of cell lines, including those of tick (IRE/CTVM19, HAE/CTVM8) and human (SH-SY5Y, WISH, SMMC, THP-1, and Vero) origin [11,15–17]. Some of these cells remain infected for an extended period (up to several months), while others exhibit cytopathic effects upon infection. The underlying mechanism of this phenotype remains unclear. Notably, other studies have been unsuccessful in culturing the virus from isolates [14,19]. The expansion of the molecular virology toolbox to include the study of segmented flaviviruses will be a crucial step in enabling a comprehensive understanding of their replication cycle and the evaluation of antiviral drugs. In particular, the development of robust and reproducible cell culture systems, including molecular clones and replicon systems, will be of great importance.

**Table 1.** Global distribution of ALSV, its host range and testing methods for genome or antibody detection.

Country	Host	Method	Reference
China	human	NS3 qPCR	[11]
China	Ixodes persulcatus	NS3 qPCR	[11]
China	Anopheles yatsushiroensis, Aedes vexans, Culex pipiens pallens, Culex tritaeniorhynchus	NS3 qPCR	[11]
China	Cattle, sheep	NS3 qPCR, VP2 ELISA	[13]
Finland	Ixodes ricinus	NGS, NS5-like qPCR	[14]
Finland	human	NS5-like qPCR, VP1a, VP1b, core IFA	[14]
France	Ixodes spp.	NGS	[22]
Russia	Ixodes persulcatus	NS5-like qPCR	[15]
Russia	Mosquitos	NS5-like qPCR	[16]
Russia	Ixodes persulcatus, D. nuttalli, H. concinna, Ixodes ricinus	NS5-like qPCR	[16]
Russia	Ixodes persulcatus	NS5-like qPCR	[17]
Germany	Ixodes spp.	NS5-like qPCR	[19]
Germany	Wild boar, Roe Deer, Red deer	NS5-like qPCR, Anti-core LIPS	[19]
Switzerland	Ixodes ricinus	NGS	[20]
Switzerland	Ixodes spp.	NS3 qPCR	[21]
China	Reindeer	NS5-like qPCR, VP2 ELISA	[69]
China	Ticks	NS5-like qPCR	[69]

Note: IFA, immunofluorescence assay.

In summary, ALSV has been detected in a wide range of hosts, which is consistent with the host-range of ticks from the *Ixodidae* family that are thought to be the main reservoir of ALSV. However, with limited studies investigating ALSV distribution using different methodologies, the overall prevalence remains difficult to estimate. It is recommended that future ALSV prevalence assessments be complemented by a standardized methodology that includes reference sequences and commercial kits for the detection and quantification of nucleic acids and antibodies. In addition, these could be complemented by pan-Jingmenvirus detection methods, which could provide additional insight into the diversity of these viruses. In addition, it may be relevant to examine sentinel animals that have regular contact with ticks, which can provide valuable information on the presence of ALSV.

## Genetic diversity of Alongshan virus

Despite the fact that flaviviruses display differences in host reservoirs, species tropism, and genetic composition, members of this family have been traditionally believed to have a similar genome structure. This positive sense ssRNA genome encodes a single open reading frame (ORF) and lacks a polyadenylation signal. The encoded ORF is translated as single polyprotein which is post-translationally cleaved into structural and non-structural (NS) proteins. After the discovery of JMTV in 2014, this paradigm has become obsolete. The ALSV genome, like the majority of viruses in the Jingmenvirus group, is organized in four different polyadenylated segments (S1–S4, see Figure 1(b), Box 1) with similar untranslated regions [6]. Although most segmented flavi-like viruses, including ALSV, share this genomic structure, others – e.g. Guaico Culex virus – possess 5 genome segments and lack polyadenylation [26]. Despite these differences in genome architecture, ALSV segment 1 and 3 encoded proteins share high degrees of homology with NS5 and NS3 of prototypic flaviviruses, respectively [11]. Segment 1 encodes a NS5-like protein sharing 26% identical (and 52% chemically similar) amino acids with a 814 amino acid stretch of the yellow fever virus (YFV) NS5 RNA-dependent RNA polymerase (RdRp) (Figure 1(b)) [7]. Despite relatively low sequence similarity, structural and functional homology of the NS5 domain between ALSV and canonical flaviviruses has been demonstrated. This includes its three-dimensional protein folding and the substrate binding properties of the methyltransferase domain [27]. Segment 3 encodes a NS3-like protein with 23% amino acid homology (and 52% chemical amino acid similarity) to a 508 amino acid segment within YFV NS3 serine protease and helicase domains [7]. Similarly, despite the low level of sequence homology, the overall structure, including the ATPase active site and the RNA binding groove, appears to be conserved between ALSV and canonical flaviviruses [28]. Since the enzymatic function of the methyltransferase, RdRp, protease or helicase is essential for flaviviral replication, these proteins may facilitate the possibility of antiviral drug design or drug repurposing. Segment 2 and 4 encoded proteins show fewer similarities with the counterparts of prototypical flaviviruses and appear to be more distantly related to them. They also appear to diverge within the Jingmenvirus group, particularly segment 2, which encodes envelope glycoproteins of Jingmenviruses. In JMTV, these glycoproteins are encoded by a single ORF (VP1), whereas in ALSV they are bicistronic, encoding the glycoproteins VP1a and VP1b (Figure 1(b)). As these glycoproteins initially showed no apparent homology to typical flaviviruses envelope proteins or those of other viruses, as determined by BLASTx or BLASTp, it was initially assumed that they were unrelated. More recently, however, Garry and Garry et al. have shown that a small region of 190 amino acids within these glycoproteins resembles other flaviviruses, containing 23% identical and 50% chemically similar amino acids [7]. In addition, they suggested that the glycoproteins of viruses from the Jingmenvirus group have diverged from a common class II viral fusion protein ancestor. As these divergent evolutionary signatures exist both between prototype flaviviruses and the Jingmenvirus group, as well as within the Jingmenvirus group, new questions arise about their evolutionary history, cell tropism, and mode of infection. The fourth segment (S4) encodes the viral capsid protein, which has no close homology to any proteins of other flaviviruses. This segment also encodes viral protein 3 (VP3), a predicted membrane protein who’s function is currently unknown. Between JMTV and ALSV, S4 is relatively well conserved with ∼75% similarity at the amino acid level. Although some proteins exhibit high conservation between ALSV and other members of the Jingmenvirus group as well as canonical flaviviruses, our understanding of the cellular processes engaged in ALSV replication or pathology remains limited. The ectopic expression of ALSV proteins demonstrated similarities in the cellular localization of some viral proteins to the endoplasmic reticulum (ER), a key feature of many flaviviruses [29]. Moreover, ectopic expression of NSP1 was observed to diminish the quantity of mitochondria, indicating potential mechanisms for virus-induced pathology. Nevertheless, further investigation is essential to gain a deeper comprehension of the molecular mechanisms underlying Jingmen virus replication and pathology, with the aim of utilizing them for the development of novel antiviral therapeutics.

Box 1. **Alongshan virus at a glance.**

*Phylogenetic* Flavi-like virus

*classification:* Related to *Orthoflavivirus*, but

     remains as unclassified

     *Jingmenvirus group*

*Genome* ssRNA, 4 Segments (S1 - S4)

  S1: NS5-like RdRp, 2988 bp

  S2: Glycoproteins, 2752 bp

  S3: NS3-like protease, 2721 bp

  S4: Core and VP3, 2701 bp

*Host range* Arthropods and mammals

*Virion* Spherical, 80–100 nm,

  enveloped

*Symptoms* Fever, headaches, fatigue

*Treatment* no specific treatment

  ribavirin (off-label)

**Figure 2.**
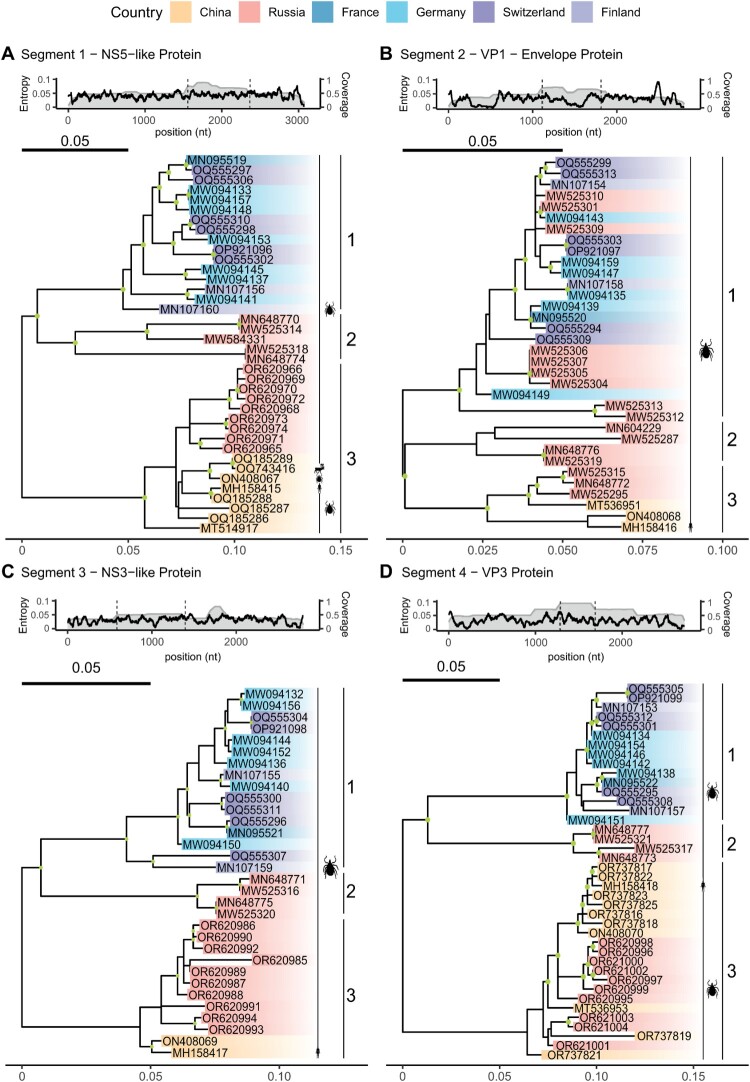
Phylogenetic analysis of ALSV genome segments. (a) segment 1, (b) segment 2, (c) segment 3 and (d) segment 4. Phylogenetic analysis was conducted using publicly available data downloaded from NCBI (n = 272, Table S1). For each segment, Shannon entropy and alignment coverage was calculated (top panel). Partial nucleotide sequences (n = 144) from the highlighted area (dotted line) were aligned using clustal omega and subsequently used to construct phylogenetic trees. Trees (below) were generated using the maximum likelihood method implemented in IQtree2 with the best model finder option. Values assigned to deep internal nodes (green circles) within the phylogeny represent bootstrap supports above 80 from 1,000 repetitions. Each tree was normalized to the scale of 0.05 mutations per site. Branch colour highlighting indicates sample origin. Icons denoted at the tips indicate host species. Deep divergences within the tree resulted in three generally well supported clusters denoted as C1, C2, C3. Within these, C1 derived sequences originated from Europe, C2 sequences from middle Asia and C3 from eastern parts of Asia. Icons have been retrieved from phylopic.org.

Few studies have investigated the global sequence diversity of ALSV and other Jingmenviruses. Virus genome sequences were uploaded from samples collected in China, Russia, France, Germany, Switzerland and Finland – a total of 272 sequences have been deposited online as of yet (Figure 2, Table S1). To assess genomic diversity for all segments, we calculated Shannon entropy (Figure 2, top panel). No apparent differences in viral diversity or mutational hotspots were observed between the downloaded sequences. Followingly, maximum likelihood phylogeny for each segment was generated from these sequences after filtering (Figure 2). Each segment followed similar phylogenetic clustering, suggesting that genetic relatedness is correlated with geographic region of sampling, rather than, for example, infection of different host organisms. Consistent with this, viral genomes sampled from the same geographical region have been reported to have high sequence similarity (>90%) to each other [11]. In contrast, genomes from geographically distant regions are more divergent. This leads to a clustering of genomes into three defined clades, which is consistent for each segment: Clade 1 represents sequences sampled mainly in or near Europe, Clade 2 represents sequences sampled around the Russia-Kazakhstan border, and Clade 3 represents sequences from the eastern part of Asia sampled from the Russian-Mongolian border (Figure 2). This evolutionary pattern would be consistent with the diversification of JMTV [30] and other tick-borne viruses such as Tick Borne Encephalitis Virus (TBEV), which diversified into a European, Siberian, and East Asian cluster [31]. However, given the limited amount of sequence data available, these patterns might be expanded in the future. Furthermore, inter – or intra host evolution of these viruses remains to be determined, including their ability to recombine or acquire mutations that promote circulation in the human population. Understanding this will be important for assessing the risk of spillover events, monitoring future outbreaks of ALSV or related viruses, and developing diagnostic tools.

## Alongshan virus and human health

To date, the incidence and prevalence of tick-borne ALSV infection in humans remains difficult to estimate, as data on human infections have only been collected in China [11]. However, the fact that ALSV has been detected in tick populations in close proximity to humans is a good example of the consequences of urbanization and raises concerns about future outbreaks. Indeed, a vast majority of the 86 patients that acquired ALSV infection in China had been working outdoors in fields, forests, or agriculture prior to the tick bite and the incubation period until onset of disease spanned from one to 10 days. Infection with ALSV in these patients caused febrile illness with unspecific symptoms, resembling infections caused by other vector-transmitted Jingmenviruses [11]. The most common clinical symptoms were fever, headaches, and fatigue, followed by other non-specific symptoms such as depression, coma, nausea, myalgia/arthralgia, and rashes. With limited knowledge on ALSV infection, however, clinical diagnosis alone can prove to be challenging and requires a combination of more extensive testing methods. These include measurements of routine laboratory parameters such as lactate dehydrogenase (LDH), C-reactive protein (CRP), creatine kinase (CK), and liver transaminases, as well as measurements of cerebrospinal fluid (CSF) parameters. In infected patients, elevated levels of C-reactive protein and lactate dehydrogenase were observed in 50% and 68%, respectively, while elevated liver transaminases were observed in 25-29% of cases and creatine kinase levels were elevated in 9% of cases [11]. Radiological imaging revealed ischemic white matter demyelination in the brain in 7 out of 54 (13%) cases and one patient showed elevated protein and white-cell count in the CSF. Additionally, serological analyses in the active and convalescent phases of infection have also been performed to detect human ALSV infection using immunofluorescence and microneutralization assays, where seroconversion was defined as at least fourfold increased levels of virus-specific IgG in the convalescent phase. Furthermore, nucleic acid amplification (RT–PCR) from whole-blood samples using virus-specific primers has been used to detect ALSV virus infection in human patients. Although the circulation of ALSV at the human tick interface is still poorly understood, methods to detect this emerging pathogen should be improved to control future outbreaks.

None of the symptomatic ALSV infections resulted in long-term damage or death. The clinical symptoms resolved within 6–8 days under empirical treatment with ribavirin and benzylpenicillin and the duration of hospitalization ranged from 10 to 14 days [11]. So far, no proof of transmission between ALSV-infected and non-infected humans exists. However, vector-borne flaviviruses have in the past caused epidemics with significant public health impact [32–34]. Therefore, the development and implementation of sufficiently effective preventative measures such as vector monitoring, diagnostic tools, and therapeutic strategies, are essential to contain potential future outbreaks/epidemics. This includes surveillance of circulating strains, research on the molecular properties of emerging virus groups, the administration of vaccines or antiviral drugs, as well as other disease-specific measures, which have been undertaken to help combat the vector-borne flavivirus disease burden [35–37]. Moreover, medical information, public education and awareness play a significant part in the spread and control of vector-borne disease.

The clinical presentation of ALSV infection is reminiscent of other tick-borne viruses causing nonspecific febrile disease. While a clear tissue tropism of ALSV infection has not yet been established, the documented systemic symptoms including fever, headache, fatigue, coma, and nausea might imply a central nervous-tropism. Therefore, it could be speculated that ALSV possesses properties and virulence factors similar to tick-borne flaviviruses which cause encephalitic disease. Tick-borne flaviviruses usually present in one of two general phenotypes: haemorrhagic fever or encephalitic disease. The pathogenicity of TBE viruses in humans, including their neuroinvasiveness and neurovirulence, is mediated by virulence factors including structural proteins, non-structural proteins, and untranslated regions (UTRs) of the viral mRNA, among others [38]. These factors play a role in determining processes such as cell entry, replication, as well as antigenicity and immune evasion. The NS5 protein of TBEV, e.g. has been linked to the development of neurological disease phenotypes of the virus in mice [39]. At present, the extent to which such factors may also be relevant for Alongshan virus is still unclear and requires further investigation. Similar to other flaviviruses, however, ALSV appears to employ strategies to circumvent host defence mechanisms, e.g. by interfering with IFN-β-mediated ISG induction through degradation of STAT2 [40]. Identification of pathogenic mechanisms, including host-virus interactions, will be crucial for understanding and combating ALSV infections in humans.

## Influences of global warming and urbanization on viral spread

**Figure 3.**
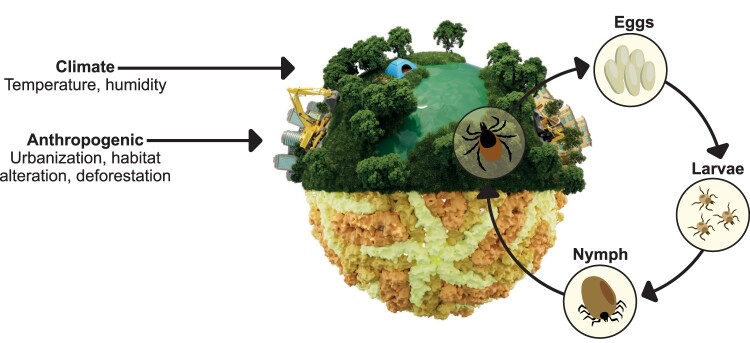
Tick-borne virus-human interface. Climate change and urbanization may increase the vector-human interface in the future. Potential interfaces include urban green spaces, parks, suburban areas and outdoor activities. Ticks can multiply in these and potentially transmit diseases.

Previous research has highlighted the fact that successful vector-mediated pathogen transmission requires the simultaneous presence of vector, pathogen and vertebrate host organism in a given space [41]. The vector-host interface therefore plays an important role in modulating the probability of pathogen transmission. This interface has received increased attention in recent years, as pathogen spillover from wildlife has been reported for many viruses leading to large socioeconomic impacts on human populations. In addition, its impact increases with changes in land use due to climate change or human encroachment on wildlife habitats (depicted in Figure 3). For instance, weather (short-term) or climate (long-term) conditions affect vegetation, biodiversity, host abundance, and vector reproduction and must allow pathogen replication within the vector in order for pathogen transmission to occur. Changes of abiotic factors such as climate have been directly tied to pathogen transmission dynamics before, demonstrating, for example, that warm temperature anomalies affect West Nile Virus transmission via mosquitoes in North America [42]. Ticks, another major carrier for a multitude of vector-borne infectious agents, and their survival, development, questing behaviour, and pathogen transmission are implicated by temperature fluctuations, with warmer and more humid conditions tending to be more favourable [43,44]. Climate change may therefore promote the geographical redistribution of ticks, leading to their emergence in previously unaffected regions with naive populations. A recent example of this is the detection of Crimean-Congo haemorrhagic fever virus RNA in southern France [45]. Changes in development, behavior, and geographical spread of Ixodes ticks, the main vector for Lyme disease and tick-borne encephalitis in Europe, have been linked to climate change, globalization and other aforementioned factors, leading to an increase of vector-borne disease incidence of Lyme Borreliosis and tick-borne encephalitis (TBE) [46–51]. Other viruses of the TBEV serocomplex, such as Powassan virus (POWV), another tick-borne pathogen causing encephalopathy in humans, have seen an increase in cases in recent years, although the exact underlying causes remain uncertain. As with other TBEVs, however, climate change and anthropogenic factors are being suggested as contributing forces [52]. Due to the shared vector type, it is plausible that Alongshan virus distribution is affected by the same dynamics, which may in turn affect epidemiology and risk of ALSV infection in the future. Thus, on a larger scale, climate change represents an important driver of increased emergence of vector-borne diseases in the future [53,54]. However, non-climate-related elements also play a substantial role in the spread of vector-borne diseases including anthropogenic factors that greatly affect native ecosystems [55,56]. Habitat alterations caused by population growth, agriculture, de – and reforestation, and urbanization affect distribution of vectors and change vector-host-overlap, causing shifts in the epidemiology of vector-borne infectious diseases [57–59]. Additionally, human movement and behaviour, including on an individual and on a population level, affect exposure to ticks and, therefore, pathogen transmission rates [60]. One consequence of this is a growing incidence of infectious diseases across an increasingly urbanized world and it appears that the combination of these factors may even outrank climate change as a driving force for the emergence of vector-borne diseases [61].

Particularly, across an urban-to-rural gradient, landscape configuration and habitat connectivity, e.g. of urban green spaces, have been shown to directly impact tick abundance and tick-borne disease incidence through alteration of the population, behaviour, or physiology of ticks and their host species [62–64]. For example, Ixodes tick parasitism has been demonstrated to be reduced in urban bird populations in eastern France [65]. While most abundant in rural areas and significantly reduced numbers in sampling sites around urbanized areas in Sweden, Ixodes ricinus presence could also be recorded in green spaces within highly urbanized areas, albeit to a lesser degree [66]. Host species that are able to adapt to the increasingly fragmented, urbanized environments represent important players in tick maintenance and pathogen reservoirs and increase transmission risk of tick-borne disease to humans in these otherwise less-favourable environments [67].

In addition to bacterial pathogens, *Flaviviridae* appear among the most prominent infectious agents transmitted by the two key vector types, mosquitoes and ticks [61]. The newly emerging, segmented flavi-like viruses have attracted particular interest lately. The epidemiology and risk of infection of ALSV are likely to be affected by the same mechanisms, similar to other tick-borne viruses such as TBEV [51,68]. Understanding these complex dynamics of anthropogenic land use changes and the influence on the tick-host-predator system is, therefore, crucial for risk assessment and reduction of emerging tick bite-associated diseases, including ALSV and similar vector-borne viruses.

## Concluding remarks

Due to climate change, globalization and urbanization, emerging viruses present a constant threat to global health. In particular, spill overs of viruses that are transmitted through arthropod vectors are becoming more frequent as the wildlife human interface increases, highlighting the significance to human health. The emergence of evolutionary distinct viruses, such as ALSV, presents a new and understudied risk. Therefore, it is crucial to examine the genomic diversity and prevalence of these viruses to prepare for potential spill overs that may occur in the near future and establish preventive measures.

## Supplementary Material

20240724_ALSVreviewSupplements.docx
