# Supplementary material for: Epidemiology and global spread of emerging tick-borne Alongshan virus
Source: Emerg Microbes Infect. 2024 Sep 11;13(1):2404271. doi: 10.1080/22221751.2024.2404271 (PMC11423535; doi:10.1080/22221751.2024.2404271)
Supplement: 20240724_ALSVreviewSupplements.docx [file TEMI_A_2404271_SM3434.docx]

**Supplementary data**

**Epidemiology and global spread of emerging tick-borne Alongshan virus**

André Gömer^1*^, Arthur Lang^2*^, Saskia Janshoff^1,4^, Joerg Steinmann^2,3^, Eike Steinmann^1*^

*1 Department for Molecular und Medical Medicine, Ruhr University Bochum, Germany*

*2 Institute of Clinical Hygiene, Medical Microbiology and Infectiology, General Hospital Nürnberg, Paracelsus Medical University, Nuremberg, Germany*

*3 Institute of Medical Microbiology, University Hospital of Essen, Essen, Germany*

*4 Institut für Laboratoriums- und Transfusionsmedizin, Herz- und Diabeteszentrum Nordrhein-Westfalen, Universitätsklinik der Ruhr-Universität Bochum, Bad Oeynhausen, Germany*

** These authors contributed equally to this work and share first/last authorship.*

**Correspondence**: Eike Steinmann ([eike.steinmann@rub.de](mailto:eike.steinmann@rub.de))

Table S1: Metadata of all sequences retrieved from NCBI.

| **Accessionnumber** | **Segment** | **Genome length (bp)** | **Organism** | **Host** | **Country** | **Collection_date** | **PubMed_ID** | **Taxon_ID** | **GC-%** |
| --- | --- | --- | --- | --- | --- | --- | --- | --- | --- |
| OQ743416.1 | 1 | 2745 | Alongshan virus | Rangifer tarandus | China | Jun 22 | NA | 2269360 | 55.15 |
| OQ185286.1 | 1 | 2745 | Alongshan virus | Ixodes persulcatus | China: Genhe, Inner Mongolia | Jul 22 | NA | 2269360 | 54.94 |
| OQ185287.1 | 1 | 2745 | Alongshan virus | Ixodes persulcatus | China: Genhe, Inner Mongolia | Jul 22 | NA | 2269360 | 55.37 |
| OQ185288.1 | 1 | 2745 | Alongshan virus | Ixodes persulcatus | China: Genhe, Inner Mongolia | Jul 22 | NA | 2269360 | 55.63 |
| OQ185289.1 | 1 | 2745 | Alongshan virus | Ixodes persulcatus | China: Genhe, Inner Mongolia | Jul 22 | NA | 2269360 | 55.19 |
| MT514916.1 | 1 | 2863 | Alongshan virus | Ixodes persulcatus | China: Heilongjiang | 2018 | NA | 2269360 | 54.56 |
| MT514917.1 | 1 | 2863 | Alongshan virus | Ixodes persulcatus | China: Heilongjiang | 2018 | NA | 2269360 | 54.56 |
| MN218594.1 | 1 | 432 | Alongshan virus | sheep | China: Hulunbuir, Inner Mongolia | 01-May-2017 | NA | 2269360 | 55.79 |
| MN218595.1 | 1 | 432 | Alongshan virus | sheep | China: Hulunbuir, Inner Mongolia | 01-May-2017 | NA | 2269360 | 56.25 |
| MN218596.1 | 1 | 432 | Alongshan virus | cattle | China: Hulunbuir, Inner Mongolia | 01-May-2017 | NA | 2269360 | 56.02 |
| MN218597.1 | 1 | 432 | Alongshan virus | cattle | China: Hulunbuir, Inner Mongolia | 01-May-2017 | NA | 2269360 | 56.71 |
| MH158440.1 | 1 | 249 | Alongshan virus | Ixodes persulcatus; WZD T52 | China: Hulunbuir, Inner Mongolia | 13-May-2017 | 31141633 | 2269360 | 54.62 |
| MH158415.1 | 1 | 2994 | Alongshan virus | Homo sapiens; WZD 3 | China: Hulunbuir, Inner Mongolia | 23-Mar-2017 | 31141633 | 2269360 | 54.38 |
| ON408067.1 | 1 | 3066 | Alongshan virus | Ixodes persulcatus WZD NE-TH4 | China: Tahe, Heilongjiang | 25. Jun 21 | NA | 2269360 | 54.60 |
| MN107160.1 | 1 | 3014 | Alongshan virus | Ixodes ricinus | Finland | 2011 | 31290392 | 2269360 | 54.28 |
| MN107156.1 | 1 | 2988 | Alongshan virus | Ixodes ricinus | Finland | 2017 | 31290392 | 2269360 | 54.15 |
| MN095519.1 | 1 | 2992 | Jingmen tick virus | Ixodes ricinus | France: Alsace | NA | NA | 1491393 | 53.44 |
| MW094133.1 | 1 | 3076 | Harz mountain virus | Ixodes sp. | Germany | 2018 | NA | 2925819 | 54.10 |
| MW094137.1 | 1 | 3076 | Harz mountain virus | Ixodes sp. | Germany | 2018 | NA | 2925819 | 54.06 |
| MW094141.1 | 1 | 3076 | Harz mountain virus | Ixodes sp. | Germany | 2018 | NA | 2925819 | 53.93 |
| MW094145.1 | 1 | 3076 | Harz mountain virus | Ixodes sp. | Germany | 2018 | NA | 2925819 | 54.32 |
| MW094148.1 | 1 | 3076 | Harz mountain virus | Ixodes sp. | Germany | 2018 | NA | 2925819 | 54.10 |
| MW094153.1 | 1 | 3076 | Harz mountain virus | Ixodes sp. | Germany | 2019 | NA | 2925819 | 54.03 |
| MW094157.1 | 1 | 3076 | Harz mountain virus | Ixodes sp. | Germany | 2019 | NA | 2925819 | 54.10 |
| MW556738.1 | 1 | 187 | Alongshan virus | Haemaphysalis concinna | Russia: Altay region | 2016 | NA | 2269360 | 49.73 |
| MW556739.1 | 1 | 187 | Alongshan virus | Haemaphysalis concinna | Russia: Altay region | 2016 | NA | 2269360 | 51.34 |
| MN648770.2 | 1 | 3085 | Alongshan virus | Ixodes persulcatus | Russia: Chelyabinsk region | May-2014 | 32224888 | 2269360 | 53.94 |
| MN648774.2 | 1 | 3074 | Alongshan virus | Ixodes persulcatus | Russia: Chelyabinsk region | May-2014 | 32224888 | 2269360 | 54.36 |
| MT210218.1 | 1 | 767 | Alongshan virus | Ixodes persulcatus | Russia: Chelyabinsk region | May-2014 | 32224888 | 2269360 | 57.37 |
| MT210219.1 | 1 | 153 | Alongshan virus | Ixodes persulcatus | Russia: Chelyabinsk region | May-2014 | 32224888 | 2269360 | 54.90 |
| MT210220.1 | 1 | 195 | Alongshan virus | Ixodes persulcatus | Russia: Chelyabinsk region | May-2014 | 32224888 | 2269360 | 51.79 |
| MT210221.1 | 1 | 195 | Alongshan virus | Ixodes persulcatus | Russia: Chelyabinsk region | May-2014 | 32224888 | 2269360 | 52.31 |
| MT210222.1 | 1 | 195 | Alongshan virus | Ixodes persulcatus | Russia: Chelyabinsk region | May-2014 | 32224888 | 2269360 | 51.79 |
| MT210223.1 | 1 | 195 | Alongshan virus | Ixodes persulcatus | Russia: Chelyabinsk region | May-2014 | 32224888 | 2269360 | 52.31 |
| MT210224.1 | 1 | 194 | Alongshan virus | Ixodes persulcatus | Russia: Chelyabinsk region | May-2014 | 32224888 | 2269360 | 51.55 |
| MT210225.1 | 1 | 195 | Alongshan virus | Ixodes persulcatus | Russia: Chelyabinsk region | May-2014 | 32224888 | 2269360 | 51.79 |
| MW525314.2 | 1 | 2975 | Alongshan virus | Ixodes persulcatus | Russia: Chelyabinsk region | May-2014 | 33799742 | 2269360 | 54.02 |
| MW525318.1 | 1 | 2975 | Alongshan virus | Ixodes persulcatus | Russia: Chelyabinsk region | May-2014 | 33799742 | 2269360 | 54.49 |
| ON448346.1 | 1 | 128 | Alongshan virus | Ixodes persulcatus | Russia: Chelyabinsk region | May-2014 | NA | 2269360 | 43.75 |
| ON448347.1 | 1 | 562 | Alongshan virus | Ixodes persulcatus | Russia: Chelyabinsk region | May-2014 | NA | 2269360 | 53.20 |
| OR620973.1 | 1 | 813 | Alongshan virus | Ixodes persulcatus | Russia: Irkutsk Oblast | 2023 | NA | 2269360 | 55.47 |
| OR620974.1 | 1 | 813 | Alongshan virus | Ixodes persulcatus | Russia: Irkutsk Oblast | 2023 | NA | 2269360 | 55.47 |
| OQ789396.1 | 1 | 224 | Alongshan virus | Ixodes ricinus | Russia: Kaliningrad Region | 2017 | 37112951 | 2269360 | 53.57 |
| MW556740.1 | 1 | 187 | Alongshan virus | Dermacentor nuttalli | Russia: Republic Altay | 2016 | NA | 2269360 | 51.34 |
| MW556741.1 | 1 | 187 | Alongshan virus | Dermacentor nuttalli | Russia: Republic Altay | 2016 | NA | 2269360 | 51.34 |
| OR620966.1 | 1 | 813 | Alongshan virus | Ixodes persulcatus | Russia: Republic of Khakassia | 2023 | NA | 2269360 | 56.09 |
| OR620967.1 | 1 | 813 | Alongshan virus | Ixodes persulcatus | Russia: Republic of Khakassia | 2023 | NA | 2269360 | 55.84 |
| OR620968.1 | 1 | 813 | Alongshan virus | Ixodes persulcatus | Russia: Republic of Khakassia | 2023 | NA | 2269360 | 55.47 |
| OR620969.1 | 1 | 813 | Alongshan virus | Ixodes persulcatus | Russia: Republic of Khakassia | 2023 | NA | 2269360 | 55.84 |
| OR620970.1 | 1 | 813 | Alongshan virus | Ixodes persulcatus | Russia: Republic of Khakassia | 2023 | NA | 2269360 | 55.84 |
| OR620971.1 | 1 | 813 | Alongshan virus | Ixodes persulcatus | Russia: Republic of Tuva | 2023 | NA | 2269360 | 55.10 |
| OR620972.1 | 1 | 813 | Alongshan virus | Ixodes persulcatus | Russia: Republic of Tuva | 2023 | NA | 2269360 | 55.72 |
| MW525296.1 | 1 | 713 | Alongshan virus | Ixodes persulcatus | Russia: Republic of Tuva | May-2017 | 33799742 | 2269360 | 56.10 |
| MW584331.1 | 1 | 2312 | Alongshan virus | Ixodes persulcatus | Russia: The Republic of Karelia | Jun 14 | 33799742 | 2269360 | 55.06 |
| OR620965.1 | 1 | 813 | Alongshan virus | Ixodes persulcatus | Russia: Zabaykalsky Krai | 2023 | NA | 2269360 | 55.23 |
| OQ555306.1 | 1 | 2935 | Alongshan virus | Ixodes ricinus | Switzerland: Grisons | May-2021 | NA | 2269360 | 53.97 |
| OP921096.1 | 1 | 3028 | Alongshan virus | Ixodes ricinus | Switzerland: Grisons | May-2022 | 36779723 | 2269360 | 53.73 |
| OQ555302.1 | 1 | 2943 | Alongshan virus | Ixodes ricinus | Switzerland: Grisons | May-2022 | NA | 2269360 | 53.69 |
| OQ555310.1 | 1 | 3022 | Alongshan virus | Ixodes ricinus | Switzerland: Schaffhausen | Sep 22 | NA | 2269360 | 54.24 |
| OQ555298.1 | 1 | 2929 | Alongshan virus | Ixodes ricinus | Switzerland: Schaffhausen | May-2021 | NA | 2269360 | 53.94 |
| OQ555297.1 | 1 | 2893 | Alongshan virus | Ixodes ricinus | Switzerland: Schaffhausen | May-2022 | NA | 2269360 | 54.27 |
| OR855394.1 | 2 | 352 | Alongshan virus | Homo sapiens | China | Apr 22 | NA | 2269360 | 56.25 |
| OR855395.1 | 2 | 361 | Alongshan virus | Homo sapiens | China | Apr 22 | NA | 2269360 | 56.23 |
| OQ716542.1 | 2 | 373 | Alongshan virus | tick | China | 01. Aug 22 | NA | 2269360 | 52.28 |
| OR855396.1 | 2 | 382 | Alongshan virus | Homo sapiens | China | Apr 23 | NA | 2269360 | 56.54 |
| OR855397.1 | 2 | 382 | Alongshan virus | Homo sapiens | China | Apr 23 | NA | 2269360 | 56.54 |
| OR855398.1 | 2 | 382 | Alongshan virus | Homo sapiens | China | Apr 23 | NA | 2269360 | 56.54 |
| OR855406.1 | 2 | 382 | Alongshan virus | Homo sapiens | China | Jun 23 | NA | 2269360 | 56.54 |
| OR855407.1 | 2 | 382 | Alongshan virus | Homo sapiens | China | Jun 23 | NA | 2269360 | 56.54 |
| OR855408.1 | 2 | 382 | Alongshan virus | Homo sapiens | China | Jun 23 | NA | 2269360 | 57.07 |
| OR855409.1 | 2 | 382 | Alongshan virus | Homo sapiens | China | Jun 23 | NA | 2269360 | 56.02 |
| OR855410.1 | 2 | 382 | Alongshan virus | Homo sapiens | China | Jun 23 | NA | 2269360 | 56.54 |
| OR855411.1 | 2 | 382 | Alongshan virus | Homo sapiens | China | Jun 23 | NA | 2269360 | 56.54 |
| OR855412.1 | 2 | 382 | Alongshan virus | Homo sapiens | China | Jun 23 | NA | 2269360 | 56.81 |
| OR855413.1 | 2 | 382 | Alongshan virus | Homo sapiens | China | Jul 23 | NA | 2269360 | 56.54 |
| OR855399.1 | 2 | 382 | Alongshan virus | Homo sapiens | China | May-2023 | NA | 2269360 | 56.81 |
| OR855400.1 | 2 | 382 | Alongshan virus | Homo sapiens | China | May-2023 | NA | 2269360 | 56.54 |
| OR855401.1 | 2 | 382 | Alongshan virus | Homo sapiens | China | May-2023 | NA | 2269360 | 56.54 |
| OR855402.1 | 2 | 382 | Alongshan virus | Homo sapiens | China | May-2023 | NA | 2269360 | 56.81 |
| OR855403.1 | 2 | 382 | Alongshan virus | Homo sapiens | China | May-2023 | NA | 2269360 | 56.54 |
| OR855404.1 | 2 | 382 | Alongshan virus | Homo sapiens | China | May-2023 | NA | 2269360 | 56.81 |
| OR855405.1 | 2 | 382 | Alongshan virus | Homo sapiens | China | May-2023 | NA | 2269360 | 56.02 |
| MZ676705.1 | 2 | 2507 | Alongshan virus | Haemaphysalis longicornis | China | NA | NA | 2269360 | 56.28 |
| MT536950.1 | 2 | 2418 | Alongshan virus | Ixodes persulcatus | China: Heilongjiang | 2018 | NA | 2269360 | 55.79 |
| MT536951.1 | 2 | 2418 | Alongshan virus | Ixodes persulcatus | China: Heilongjiang | 2018 | NA | 2269360 | 55.79 |
| MH158416.1 | 2 | 2806 | Alongshan virus | Homo sapiens; WZD 3 | China: Hulunbuir, Inner Mongolia | 23-Mar-2017 | 31141633 | 2269360 | 54.21 |
| ON408068.1 | 2 | 2813 | Alongshan virus | Ixodes persulcatus WZD NE-TH4 | China: Tahe, Heilongjiang | 25. Jun 21 | NA | 2269360 | 54.89 |
| MN107158.1 | 2 | 2755 | Alongshan virus | Ixodes ricinus | Finland | 2011 | 31290392 | 2269360 | 55.10 |
| MN107154.1 | 2 | 2752 | Alongshan virus | Ixodes ricinus | Finland | 2017 | 31290392 | 2269360 | 54.94 |
| MN095520.1 | 2 | 2803 | Jingmen tick virus | Ixodes ricinus | France: Alsace | NA | NA | 1491393 | 54.44 |
| MW094135.1 | 2 | 2774 | Harz mountain virus | Ixodes sp. | Germany | 2018 | NA | 2925819 | 54.97 |
| MW094139.1 | 2 | 2774 | Harz mountain virus | Ixodes sp. | Germany | 2018 | NA | 2925819 | 54.72 |
| MW094143.1 | 2 | 2774 | Harz mountain virus | Ixodes sp. | Germany | 2018 | NA | 2925819 | 54.76 |
| MW094147.1 | 2 | 2774 | Harz mountain virus | Ixodes sp. | Germany | 2018 | NA | 2925819 | 54.83 |
| MW094149.1 | 2 | 2774 | Harz mountain virus | Ixodes sp. | Germany | 2018 | NA | 2925819 | 54.76 |
| MW094155.1 | 2 | 2774 | Harz mountain virus | Ixodes sp. | Germany | 2019 | NA | 2925819 | 54.83 |
| MW094159.1 | 2 | 2774 | Harz mountain virus | Ixodes sp. | Germany | 2019 | NA | 2925819 | 54.87 |
| OP125776.1 | 2 | 314 | Alongshan virus | Ixodes persulcatus | Russia: Chelyabinsk region | 2015 | 36560703 | 2269360 | 56.37 |
| MN648772.2 | 2 | 2795 | Alongshan virus | Ixodes persulcatus | Russia: Chelyabinsk region | May-2014 | 32224888 | 2269360 | 54.13 |
| MN648776.2 | 2 | 2783 | Alongshan virus | Ixodes persulcatus | Russia: Chelyabinsk region | May-2014 | 32224888 | 2269360 | 54.22 |
| MW525315.2 | 2 | 2791 | Alongshan virus | Ixodes persulcatus | Russia: Chelyabinsk region | May-2014 | 33799742 | 2269360 | 54.21 |
| MW525319.1 | 2 | 2592 | Alongshan virus | Ixodes persulcatus | Russia: Chelyabinsk region | May-2014 | 33799742 | 2269360 | 55.17 |
| ON448348.1 | 2 | 404 | Alongshan virus | Ixodes persulcatus | Russia: Chelyabinsk region | May-2014 | NA | 2269360 | 50.50 |
| ON448349.1 | 2 | 431 | Alongshan virus | Ixodes persulcatus | Russia: Chelyabinsk region | May-2014 | NA | 2269360 | 47.80 |
| MW525284.1 | 2 | 293 | Alongshan virus | Ixodes persulcatus | Russia: Chelyabinsk region | May-2015 | 33799742 | 2269360 | 57.34 |
| MW525285.1 | 2 | 293 | Alongshan virus | Ixodes persulcatus | Russia: Chelyabinsk region | May-2015 | 33799742 | 2269360 | 56.66 |
| OR620983.1 | 2 | 998 | Alongshan virus | Ixodes persulcatus | Russia: Irkutsk Oblast | 2023 | NA | 2269360 | 54.31 |
| OR620984.1 | 2 | 998 | Alongshan virus | Ixodes persulcatus | Russia: Irkutsk Oblast | 2023 | NA | 2269360 | 54.21 |
| OQ789397.1 | 2 | 400 | Alongshan virus | Ixodes ricinus | Russia: Kaliningrad Region | 2017 | 37112951 | 2269360 | 49.00 |
| MW525297.1 | 2 | 293 | Alongshan virus | Ixodes ricinus | Russia: Kaliningrad region | Aug 17 | 33799742 | 2269360 | 57.68 |
| MW525298.1 | 2 | 293 | Alongshan virus | Ixodes ricinus | Russia: Kaliningrad region | Aug 17 | 33799742 | 2269360 | 56.66 |
| MW525299.1 | 2 | 293 | Alongshan virus | Ixodes ricinus | Russia: Kaliningrad region | Aug 17 | 33799742 | 2269360 | 58.70 |
| MW525300.1 | 2 | 722 | Alongshan virus | Ixodes ricinus | Russia: Kaliningrad region | Aug 17 | 33799742 | 2269360 | 56.51 |
| MW525310.1 | 2 | 2599 | Alongshan virus | Ixodes ricinus | Russia: Kaliningrad region | Apr 19 | 33799742 | 2269360 | 55.41 |
| MW525301.1 | 2 | 733 | Alongshan virus | Ixodes ricinus | Russia: Kaliningrad region | May-2018 | 33799742 | 2269360 | 56.34 |
| MW525302.1 | 2 | 293 | Alongshan virus | Ixodes ricinus | Russia: Kaliningrad region | May-2018 | 33799742 | 2269360 | 58.02 |
| MW525303.1 | 2 | 522 | Alongshan virus | Ixodes ricinus | Russia: Kaliningrad region | May-2018 | 33799742 | 2269360 | 56.51 |
| MW525304.1 | 2 | 732 | Alongshan virus | Ixodes ricinus | Russia: Kaliningrad region | May-2018 | 33799742 | 2269360 | 55.87 |
| MW525305.1 | 2 | 732 | Alongshan virus | Ixodes ricinus | Russia: Kaliningrad region | May-2018 | 33799742 | 2269360 | 56.15 |
| MW525306.1 | 2 | 2502 | Alongshan virus | Ixodes ricinus | Russia: Kaliningrad region | May-2018 | 33799742 | 2269360 | 55.60 |
| MW525307.1 | 2 | 714 | Alongshan virus | Ixodes ricinus | Russia: Kaliningrad region | May-2018 | 33799742 | 2269360 | 56.58 |
| MW525308.1 | 2 | 643 | Alongshan virus | Ixodes ricinus | Russia: Kaliningrad region | May-2018 | 33799742 | 2269360 | 55.68 |
| MW525309.1 | 2 | 2536 | Alongshan virus | Ixodes ricinus | Russia: Kaliningrad region | May-2018 | 33799742 | 2269360 | 55.84 |
| MW525286.1 | 2 | 293 | Alongshan virus | Ixodes persulcatus | Russia: Republic of Karelia | Jun 12 | 33799742 | 2269360 | 57.00 |
| MW525287.1 | 2 | 2593 | Alongshan virus | Ixodes persulcatus | Russia: Republic of Karelia | Jun 13 | 33799742 | 2269360 | 54.80 |
| MW525288.1 | 2 | 293 | Alongshan virus | Ixodes persulcatus | Russia: Republic of Karelia | Jun 13 | 33799742 | 2269360 | 57.00 |
| MW525289.1 | 2 | 293 | Alongshan virus | Ixodes persulcatus | Russia: Republic of Karelia | Jun 13 | 33799742 | 2269360 | 56.66 |
| MW525290.1 | 2 | 293 | Alongshan virus | Ixodes persulcatus | Russia: Republic of Karelia | Jun 13 | 33799742 | 2269360 | 57.34 |
| MW525291.1 | 2 | 293 | Alongshan virus | Ixodes persulcatus | Russia: Republic of Karelia | Jun 14 | 33799742 | 2269360 | 54.95 |
| MW525292.1 | 2 | 293 | Alongshan virus | Ixodes persulcatus | Russia: Republic of Karelia | Jun 18 | 33799742 | 2269360 | 55.97 |
| MW525293.1 | 2 | 293 | Alongshan virus | Ixodes persulcatus | Russia: Republic of Karelia | Jun 18 | 33799742 | 2269360 | 55.97 |
| MW525294.1 | 2 | 293 | Alongshan virus | Ixodes persulcatus | Russia: Republic of Karelia | Jun 18 | 33799742 | 2269360 | 54.27 |
| OR620976.1 | 2 | 998 | Alongshan virus | Ixodes persulcatus | Russia: Republic of Khakassia | 2023 | NA | 2269360 | 54.21 |
| OR620977.1 | 2 | 998 | Alongshan virus | Ixodes persulcatus | Russia: Republic of Khakassia | 2023 | NA | 2269360 | 54.41 |
| OR620978.1 | 2 | 998 | Alongshan virus | Ixodes persulcatus | Russia: Republic of Khakassia | 2023 | NA | 2269360 | 54.11 |
| OR620979.1 | 2 | 998 | Alongshan virus | Ixodes persulcatus | Russia: Republic of Khakassia | 2023 | NA | 2269360 | 54.41 |
| OR620980.1 | 2 | 998 | Alongshan virus | Ixodes persulcatus | Russia: Republic of Khakassia | 2023 | NA | 2269360 | 54.11 |
| MW525313.1 | 2 | 2447 | Alongshan virus | Ixodes ricinus | Russia: Republic of Tatarstan | Aug 14 | 33799742 | 2269360 | 55.74 |
| OR620981.1 | 2 | 998 | Alongshan virus | Ixodes persulcatus | Russia: Republic of Tuva | 2023 | NA | 2269360 | 54.31 |
| OR620982.1 | 2 | 998 | Alongshan virus | Ixodes persulcatus | Russia: Republic of Tuva | 2023 | NA | 2269360 | 54.21 |
| MW525295.1 | 2 | 2578 | Alongshan virus | Ixodes persulcatus | Russia: Republic of Tuva | May-2017 | 33799742 | 2269360 | 54.85 |
| MN604229.2 | 2 | 2651 | Alongshan virus | Ixodes persulcatus | Russia: The Republic of Karelia | Jun 14 | 32224888 | 2269360 | 54.43 |
| MW525311.1 | 2 | 230 | Alongshan virus | Dermacentor reticulatus | Russia: Ulyanovsk region | May-2014 | 33799742 | 2269360 | 57.83 |
| MW525312.1 | 2 | 2447 | Alongshan virus | Ixodes ricinus | Russia: Ulyanovsk region | May-2015 | 33799742 | 2269360 | 55.41 |
| OR620975.1 | 2 | 998 | Alongshan virus | Ixodes persulcatus | Russia: Zabaykalsky Krai | 2023 | NA | 2269360 | 54.21 |
| OQ555309.1 | 2 | 2776 | Alongshan virus | Ixodes ricinus | Switzerland: Grisons | May-2021 | NA | 2269360 | 54.43 |
| OP921097.1 | 2 | 2788 | Alongshan virus | Ixodes ricinus | Switzerland: Grisons | May-2022 | 36779723 | 2269360 | 54.70 |
| OQ555303.1 | 2 | 2675 | Alongshan virus | Ixodes ricinus | Switzerland: Grisons | May-2022 | NA | 2269360 | 54.80 |
| OQ555313.1 | 2 | 2803 | Alongshan virus | Ixodes ricinus | Switzerland: Schaffhausen | Sep 22 | NA | 2269360 | 54.12 |
| OQ555299.1 | 2 | 2695 | Alongshan virus | Ixodes ricinus | Switzerland: Schaffhausen | May-2021 | NA | 2269360 | 54.73 |
| OQ555294.1 | 2 | 2681 | Alongshan virus | Ixodes ricinus | Switzerland: Schaffhausen | May-2022 | NA | 2269360 | 54.83 |
| MH158426.1 | 3 | 159 | Alongshan virus | Homo sapiens; WZD 113 | China: Greater Khingan, Heilongjiang | 09. Apr 17 | 31141633 | 2269360 | 50.94 |
| MT246198.1 | 3 | 1531 | Alongshan virus | Ixodes persulcatus | China: Heilongjiang | 2018 | NA | 2269360 | 56.56 |
| MT246199.1 | 3 | 1531 | Alongshan virus | Ixodes persulcatus | China: Heilongjiang | 2018 | NA | 2269360 | 56.56 |
| MH158424.1 | 3 | 165 | Alongshan virus | Homo sapiens; WZD 30 | China: Hinggan, Inner Mongolia | 06. Apr 17 | 31141633 | 2269360 | 51.52 |
| MH158431.1 | 3 | 165 | Alongshan virus | Homo sapiens; WZD 217 | China: Hinggan, Inner Mongolia | 08-May-2017 | 31141633 | 2269360 | 50.91 |
| MH158434.1 | 3 | 159 | Alongshan virus | Homo sapiens; WZD 351 | China: Hinggan, Inner Mongolia | 12-May-2017 | 31141633 | 2269360 | 51.57 |
| MH158419.1 | 3 | 165 | Alongshan virus | Homo sapiens; WZD 9 | China: Hulunbuir, Inner Mongolia | 03. Apr 17 | 31141633 | 2269360 | 50.91 |
| MH158420.1 | 3 | 159 | Alongshan virus | Homo sapiens; WZD 11 | China: Hulunbuir, Inner Mongolia | 04. Apr 17 | 31141633 | 2269360 | 51.57 |
| MH158422.1 | 3 | 165 | Alongshan virus | Homo sapiens; WZD 26 | China: Hulunbuir, Inner Mongolia | 04. Apr 17 | 31141633 | 2269360 | 50.91 |
| MH158423.1 | 3 | 159 | Alongshan virus | Homo sapiens; WZD 28 | China: Hulunbuir, Inner Mongolia | 04. Apr 17 | 31141633 | 2269360 | 51.57 |
| MH158421.1 | 3 | 159 | Alongshan virus | Homo sapiens; WZD 18 | China: Hulunbuir, Inner Mongolia | 05. Apr 17 | 31141633 | 2269360 | 51.57 |
| MH158425.1 | 3 | 165 | Alongshan virus | Homo sapiens; WZD 111 | China: Hulunbuir, Inner Mongolia | 21. Apr 17 | 31141633 | 2269360 | 52.12 |
| MH158427.1 | 3 | 159 | Alongshan virus | Homo sapiens; WZD 125 | China: Hulunbuir, Inner Mongolia | 23. Apr 17 | 31141633 | 2269360 | 52.20 |
| MH158428.1 | 3 | 159 | Alongshan virus | Homo sapiens; WZD 136 | China: Hulunbuir, Inner Mongolia | 28. Apr 17 | 31141633 | 2269360 | 50.94 |
| MH158437.1 | 3 | 159 | Alongshan virus | Homo sapiens; WZD 766 | China: Hulunbuir, Inner Mongolia | 06. Jun 17 | 31141633 | 2269360 | 50.94 |
| MH158438.1 | 3 | 159 | Alongshan virus | Homo sapiens; WZD 906 | China: Hulunbuir, Inner Mongolia | 17. Jun 17 | 31141633 | 2269360 | 51.57 |
| MH158429.1 | 3 | 165 | Alongshan virus | Homo sapiens; WZD 205 | China: Hulunbuir, Inner Mongolia | 16. Jul 17 | 31141633 | 2269360 | 51.52 |
| MH158432.1 | 3 | 165 | Alongshan virus | Homo sapiens; WZD 218 | China: Hulunbuir, Inner Mongolia | 08-May-2017 | 31141633 | 2269360 | 50.30 |
| MH158433.1 | 3 | 159 | Alongshan virus | Homo sapiens; WZD 350 | China: Hulunbuir, Inner Mongolia | 10-May-2017 | 31141633 | 2269360 | 52.20 |
| MH158439.1 | 3 | 159 | Alongshan virus | Ixodes persulcatus; WZD T69 | China: Hulunbuir, Inner Mongolia | 13-May-2017 | 31141633 | 2269360 | 52.20 |
| MH158435.1 | 3 | 159 | Alongshan virus | Homo sapiens; WZD 384 | China: Hulunbuir, Inner Mongolia | 15-May-2017 | 31141633 | 2269360 | 52.83 |
| MH158417.1 | 3 | 2811 | Alongshan virus | Homo sapiens; WZD 3 | China: Hulunbuir, Inner Mongolia | 23-Mar-2017 | 31141633 | 2269360 | 55.10 |
| MH158436.1 | 3 | 159 | Alongshan virus | Homo sapiens; WZD 566 | China: Hulunbuir, Inner Mongolia | 26-May-2017 | 31141633 | 2269360 | 50.94 |
| MK122718.1 | 3 | 228 | Alongshan virus | cattle | China: Hulunbuir, Inner Mongolia | May-2017 | NA | 2269360 | 51.75 |
| MK122719.1 | 3 | 228 | Alongshan virus | sheep | China: Hulunbuir, Inner Mongolia | May-2017 | NA | 2269360 | 51.75 |
| MK122720.1 | 3 | 228 | Alongshan virus | cattle | China: Hulunbuir, Inner Mongolia | May-2017 | NA | 2269360 | 52.19 |
| MK122721.1 | 3 | 228 | Alongshan virus | sheep | China: Hulunbuir, Inner Mongolia | May-2017 | NA | 2269360 | 53.07 |
| MK213941.1 | 3 | 228 | Alongshan virus | Anopheles yatsushiroensis | China: Jilin | 01. Jul 17 | NA | 2269360 | 52.19 |
| MK213942.1 | 3 | 228 | Alongshan virus | Culex tritaeniorhynchus | China: Jilin | 01. Jul 17 | NA | 2269360 | 51.75 |
| MH158430.1 | 3 | 165 | Alongshan virus | Homo sapiens; WZD 215 | China: Qiqihar, Heilongjiang | 08-May-2017 | 31141633 | 2269360 | 50.91 |
| ON408069.1 | 3 | 2809 | Alongshan virus | Ixodes persulcatus WZD NE-TH4 | China: Tahe, Heilongjiang | 25. Jun 21 | NA | 2269360 | 55.11 |
| MN107159.1 | 3 | 2754 | Alongshan virus | Ixodes ricinus | Finland | 2011 | 31290392 | 2269360 | 55.56 |
| MN107155.1 | 3 | 2721 | Alongshan virus | Ixodes ricinus | Finland | 2017 | 31290392 | 2269360 | 54.94 |
| MN095521.1 | 3 | 2807 | Jingmen tick virus | Ixodes ricinus | France: Alsace | NA | NA | 1491393 | 54.44 |
| MW094132.1 | 3 | 2806 | Harz mountain virus | Ixodes sp. | Germany | 2018 | NA | 2925819 | 55.27 |
| MW094136.1 | 3 | 2806 | Harz mountain virus | Ixodes sp. | Germany | 2018 | NA | 2925819 | 55.31 |
| MW094140.1 | 3 | 2806 | Harz mountain virus | Ixodes sp. | Germany | 2018 | NA | 2925819 | 54.88 |
| MW094144.1 | 3 | 2806 | Harz mountain virus | Ixodes sp. | Germany | 2018 | NA | 2925819 | 55.42 |
| MW094150.1 | 3 | 2806 | Harz mountain virus | Ixodes sp. | Germany | 2018 | NA | 2925819 | 55.52 |
| MW094152.1 | 3 | 2806 | Harz mountain virus | Ixodes sp. | Germany | 2019 | NA | 2925819 | 55.35 |
| MW094156.1 | 3 | 2806 | Harz mountain virus | Ixodes sp. | Germany | 2019 | NA | 2925819 | 55.38 |
| MN648771.2 | 3 | 2801 | Alongshan virus | Ixodes persulcatus | Russia: Chelyabinsk region | May-2014 | 32224888 | 2269360 | 55.09 |
| MN648775.2 | 3 | 2793 | Alongshan virus | Ixodes persulcatus | Russia: Chelyabinsk region | May-2014 | 32224888 | 2269360 | 55.25 |
| MW525316.2 | 3 | 2798 | Alongshan virus | Ixodes persulcatus | Russia: Chelyabinsk region | May-2014 | 33799742 | 2269360 | 55.08 |
| MW525320.1 | 3 | 2702 | Alongshan virus | Ixodes persulcatus | Russia: Chelyabinsk region | May-2014 | 33799742 | 2269360 | 55.48 |
| ON448350.1 | 3 | 148 | Alongshan virus | Ixodes persulcatus | Russia: Chelyabinsk region | May-2014 | NA | 2269360 | 40.54 |
| ON448351.1 | 3 | 450 | Alongshan virus | Ixodes persulcatus | Russia: Chelyabinsk region | May-2014 | NA | 2269360 | 49.78 |
| OR620993.1 | 3 | 811 | Alongshan virus | Ixodes persulcatus | Russia: Irkutsk Oblast | 2023 | NA | 2269360 | 56.60 |
| OR620994.1 | 3 | 811 | Alongshan virus | Ixodes persulcatus | Russia: Irkutsk Oblast | 2023 | NA | 2269360 | 56.72 |
| OQ789398.1 | 3 | 266 | Alongshan virus | Ixodes ricinus | Russia: Kaliningrad Region | 2017 | 37112951 | 2269360 | 52.26 |
| OR620986.1 | 3 | 811 | Alongshan virus | Ixodes persulcatus | Russia: Republic of Khakassia | 2023 | NA | 2269360 | 56.23 |
| OR620987.1 | 3 | 811 | Alongshan virus | Ixodes persulcatus | Russia: Republic of Khakassia | 2023 | NA | 2269360 | 56.84 |
| OR620988.1 | 3 | 811 | Alongshan virus | Ixodes persulcatus | Russia: Republic of Khakassia | 2023 | NA | 2269360 | 56.47 |
| OR620989.1 | 3 | 811 | Alongshan virus | Ixodes persulcatus | Russia: Republic of Khakassia | 2023 | NA | 2269360 | 56.84 |
| OR620990.1 | 3 | 811 | Alongshan virus | Ixodes persulcatus | Russia: Republic of Khakassia | 2023 | NA | 2269360 | 56.60 |
| OR620991.1 | 3 | 811 | Alongshan virus | Ixodes persulcatus | Russia: Republic of Tuva | 2023 | NA | 2269360 | 56.84 |
| OR620992.1 | 3 | 811 | Alongshan virus | Ixodes persulcatus | Russia: Republic of Tuva | 2023 | NA | 2269360 | 56.23 |
| OR620985.1 | 3 | 811 | Alongshan virus | Ixodes persulcatus | Russia: Zabaykalsky Krai | 2023 | NA | 2269360 | 55.86 |
| OQ555307.1 | 3 | 2734 | Alongshan virus | Ixodes ricinus | Switzerland: Grisons | May-2021 | NA | 2269360 | 55.05 |
| OP921098.1 | 3 | 2808 | Alongshan virus | Ixodes ricinus | Switzerland: Grisons | May-2022 | 36779723 | 2269360 | 54.70 |
| OQ555304.1 | 3 | 2611 | Alongshan virus | Ixodes ricinus | Switzerland: Grisons | May-2022 | NA | 2269360 | 55.50 |
| OQ555311.1 | 3 | 2776 | Alongshan virus | Ixodes ricinus | Switzerland: Schaffhausen | Sep 22 | NA | 2269360 | 55.22 |
| OQ555300.1 | 3 | 2764 | Alongshan virus | Ixodes ricinus | Switzerland: Schaffhausen | May-2021 | NA | 2269360 | 55.14 |
| OQ555296.1 | 3 | 2710 | Alongshan virus | Ixodes ricinus | Switzerland: Schaffhausen | May-2022 | NA | 2269360 | 54.98 |
| OR737816.1 | 4 | 403 | Alongshan virus | Ixodes persulcatus | China | 2022/2023 | NA | 2269360 | 57.57 |
| OR737817.1 | 4 | 403 | Alongshan virus | Ixodes persulcatus | China | 2022/2023 | NA | 2269360 | 58.56 |
| OR737818.1 | 4 | 403 | Alongshan virus | Ixodes persulcatus | China | 2022/2023 | NA | 2269360 | 57.07 |
| OR737819.1 | 4 | 403 | Alongshan virus | Ixodes persulcatus | China | 2022/2023 | NA | 2269360 | 57.07 |
| OR737820.1 | 4 | 403 | Alongshan virus | Ixodes persulcatus | China | 2022/2023 | NA | 2269360 | 57.07 |
| OR737821.1 | 4 | 403 | Alongshan virus | Ixodes persulcatus | China | 2022/2023 | NA | 2269360 | 57.32 |
| OR737822.1 | 4 | 403 | Alongshan virus | Ixodes persulcatus | China | 2022/2023 | NA | 2269360 | 58.56 |
| OR737823.1 | 4 | 403 | Alongshan virus | Ixodes persulcatus | China | 2022/2023 | NA | 2269360 | 57.82 |
| OR737824.1 | 4 | 403 | Alongshan virus | Ixodes persulcatus | China | 2022/2023 | NA | 2269360 | 58.31 |
| OR737825.1 | 4 | 403 | Alongshan virus | Ixodes persulcatus | China | 2022/2023 | NA | 2269360 | 58.31 |
| MT536952.1 | 4 | 2336 | Alongshan virus | Ixodes persulcatus | China: Heilongjiang | 2018 | NA | 2269360 | 55.31 |
| MT536953.1 | 4 | 2336 | Alongshan virus | Ixodes persulcatus | China: Heilongjiang | 2018 | NA | 2269360 | 55.31 |
| MH158418.1 | 4 | 2738 | Alongshan virus | Homo sapiens; WZD 3 | China: Hulunbuir, Inner Mongolia | 23-Mar-2017 | 31141633 | 2269360 | 54.82 |
| ON408070.1 | 4 | 2721 | Alongshan virus | Ixodes persulcatus WZD NE-TH4 | China: Tahe, Heilongjiang | 25. Jun 21 | NA | 2269360 | 55.05 |
| MN107157.1 | 4 | 2696 | Alongshan virus | Ixodes ricinus | Finland | 2011 | 31290392 | 2269360 | 55.27 |
| MN107153.1 | 4 | 2697 | Alongshan virus | Ixodes ricinus | Finland | 2017 | 31290392 | 2269360 | 54.88 |
| MN095522.1 | 4 | 2735 | Jingmen tick virus | Ixodes ricinus | France: Alsace | NA | NA | 1491393 | 54.33 |
| MW094134.1 | 4 | 2736 | Harz mountain virus | Ixodes sp. | Germany | 2018 | NA | 2925819 | 54.71 |
| MW094138.1 | 4 | 2736 | Harz mountain virus | Ixodes sp. | Germany | 2018 | NA | 2925819 | 54.93 |
| MW094142.1 | 4 | 2736 | Harz mountain virus | Ixodes sp. | Germany | 2018 | NA | 2925819 | 54.68 |
| MW094146.1 | 4 | 2736 | Harz mountain virus | Ixodes sp. | Germany | 2018 | NA | 2925819 | 54.71 |
| MW094151.1 | 4 | 2736 | Harz mountain virus | Ixodes sp. | Germany | 2018 | NA | 2925819 | 54.61 |
| MW094154.1 | 4 | 2736 | Harz mountain virus | Ixodes sp. | Germany | 2019 | NA | 2925819 | 54.71 |
| MW094158.1 | 4 | 2736 | Harz mountain virus | Ixodes sp. | Germany | 2019 | NA | 2925819 | 54.71 |
| MN648773.2 | 4 | 2744 | Alongshan virus | Ixodes persulcatus | Russia: Chelyabinsk region | May-2014 | 32224888 | 2269360 | 54.48 |
| MN648777.2 | 4 | 2732 | Alongshan virus | Ixodes persulcatus | Russia: Chelyabinsk region | May-2014 | 32224888 | 2269360 | 54.65 |
| MW525317.2 | 4 | 2736 | Alongshan virus | Ixodes persulcatus | Russia: Chelyabinsk region | May-2014 | 33799742 | 2269360 | 54.61 |
| MW525321.1 | 4 | 2741 | Alongshan virus | Ixodes persulcatus | Russia: Chelyabinsk region | May-2014 | 33799742 | 2269360 | 54.62 |
| ON448352.1 | 4 | 163 | Alongshan virus | Ixodes persulcatus | Russia: Chelyabinsk region | May-2014 | NA | 2269360 | 47.85 |
| ON448353.1 | 4 | 355 | Alongshan virus | Ixodes persulcatus | Russia: Chelyabinsk region | May-2014 | NA | 2269360 | 49.86 |
| OR621003.1 | 4 | 1181 | Alongshan virus | Ixodes persulcatus | Russia: Irkutsk Oblast | 2023 | NA | 2269360 | 57.41 |
| OR621004.1 | 4 | 1181 | Alongshan virus | Ixodes persulcatus | Russia: Irkutsk Oblast | 2023 | NA | 2269360 | 57.66 |
| OQ789399.1 | 4 | 307 | Alongshan virus | Ixodes ricinus | Russia: Kaliningrad Region | 2017 | 37112951 | 2269360 | 50.49 |
| OP244356.1 | 4 | 330 | Alongshan virus | Ixodes persulcatus | Russia: Novosibirsk | 2021 | NA | 2269360 | 57.27 |
| OP244397.1 | 4 | 276 | Alongshan virus | Ixodes persulcatus | Russia: Novosibirsk | 2021 | NA | 2269360 | 55.80 |
| OP244398.1 | 4 | 276 | Alongshan virus | Ixodes persulcatus | Russia: Novosibirsk | 2021 | NA | 2269360 | 55.43 |
| OP244399.1 | 4 | 276 | Alongshan virus | Ixodes persulcatus | Russia: Novosibirsk | 2021 | NA | 2269360 | 55.80 |
| OP244400.1 | 4 | 278 | Alongshan virus | Ixodes persulcatus | Russia: Novosibirsk | 2021 | NA | 2269360 | 55.40 |
| OP244401.1 | 4 | 332 | Alongshan virus | Ixodes persulcatus | Russia: Novosibirsk | 2021 | NA | 2269360 | 57.83 |
| OP244402.1 | 4 | 332 | Alongshan virus | Ixodes persulcatus | Russia: Novosibirsk | 2021 | NA | 2269360 | 56.63 |
| OP244412.1 | 4 | 301 | Alongshan virus | Ixodes persulcatus | Russia: Novosibirsk | 2021 | NA | 2269360 | 57.48 |
| OP244413.1 | 4 | 332 | Alongshan virus | Ixodes persulcatus | Russia: Novosibirsk | 2021 | NA | 2269360 | 57.23 |
| OP244414.1 | 4 | 276 | Alongshan virus | Ixodes persulcatus | Russia: Novosibirsk | 2021 | NA | 2269360 | 54.71 |
| OR620996.1 | 4 | 1181 | Alongshan virus | Ixodes persulcatus | Russia: Republic of Khakassia | 2023 | NA | 2269360 | 56.73 |
| OR620997.1 | 4 | 1181 | Alongshan virus | Ixodes persulcatus | Russia: Republic of Khakassia | 2023 | NA | 2269360 | 56.65 |
| OR620998.1 | 4 | 1181 | Alongshan virus | Ixodes persulcatus | Russia: Republic of Khakassia | 2023 | NA | 2269360 | 56.73 |
| OR620999.1 | 4 | 1181 | Alongshan virus | Ixodes persulcatus | Russia: Republic of Khakassia | 2023 | NA | 2269360 | 57.15 |
| OR621000.1 | 4 | 1181 | Alongshan virus | Ixodes persulcatus | Russia: Republic of Khakassia | 2023 | NA | 2269360 | 56.65 |
| OR621001.1 | 4 | 1181 | Alongshan virus | Ixodes persulcatus | Russia: Republic of Tuva | 2023 | NA | 2269360 | 57.32 |
| OR621002.1 | 4 | 1181 | Alongshan virus | Ixodes persulcatus | Russia: Republic of Tuva | 2023 | NA | 2269360 | 56.65 |
| OR620995.1 | 4 | 1181 | Alongshan virus | Ixodes persulcatus | Russia: Zabaykalsky Krai | 2023 | NA | 2269360 | 56.90 |
| OQ555308.1 | 4 | 2706 | Alongshan virus | Ixodes ricinus | Switzerland: Grisons | May-2021 | NA | 2269360 | 54.95 |
| OP921099.1 | 4 | 2744 | Alongshan virus | Ixodes ricinus | Switzerland: Grisons | May-2022 | 36779723 | 2269360 | 54.12 |
| OQ555305.1 | 4 | 2661 | Alongshan virus | Ixodes ricinus | Switzerland: Grisons | May-2022 | NA | 2269360 | 54.68 |
| OQ555312.1 | 4 | 2688 | Alongshan virus | Ixodes ricinus | Switzerland: Schaffhausen | Sep 22 | NA | 2269360 | 54.76 |
| OQ555301.1 | 4 | 2679 | Alongshan virus | Ixodes ricinus | Switzerland: Schaffhausen | May-2021 | NA | 2269360 | 54.39 |
| OQ555295.1 | 4 | 2684 | Alongshan virus | Ixodes ricinus | Switzerland: Schaffhausen | May-2022 | NA | 2269360 | 54.77 |
